# Acceptability of home fortification with multiple micro-nutrients among Sri Lankan children

**DOI:** 10.1371/journal.pone.0261516

**Published:** 2021-12-22

**Authors:** G. Liyanage, K. G. I. S. Anupama, M. L. P. Sudarshini

**Affiliations:** 1 Department of Paediatrics, Faculty of Medical Sciences, University of Sri Jayewardenepura, Colombo, Sri Lanka; 2 Department of Pharmacy, Faculty of Allied Health Sciences, University of Sri Jayewardenepura, Colombo, Sri Lanka; Addis Ababa University, ETHIOPIA

## Abstract

Micronutrient deficiencies are mostly hidden; clinically less visible compared to macronutrient deficiencies. Food fortification with multiple micronutrients (MMN) is provided for children between 6–23 months, daily for two months at three-time points. We assessed the acceptance and adherence of this nutritional intervention in an urban community setting in Sri Lanka. This cross-sectional study enrolled caregivers of children aged 7 to 23 months with a cluster sampling method. Caregivers ’ acceptance of taste and smell, health gains, ease of use, and need perception (Cronbach’s reliability: 0.801) were assessed. Also, anemia knowledge (Cronbach’s reliability: 0.642), MMN knowledge, and reported adherence (number of sachets consumed per month) were evaluated through a self-administered questionnaire. Adequate adherence was defined as the use of ≥80% sachets. The univariate and multivariate statistical analysis examined the association of acceptability, adherence, and anemia knowledge with independent variables (socio-demographic, household characteristics, and knowledge). The survey included 153 respondents. The Median (range) age of children was 12 months (7–23). The mean (SD) acceptability score was 66.82% (9.78%). Acceptance of sensory qualities (smell/taste) had a lower score than perceived health benefit. Most consumed MMN adequately (72.5%). The mean (SD) anemia knowledge score was 62.20% (25.79%). In multivariate analysis, child’s age (OR: -0.360, 95% CI:-0.510,-0.211) and father’s education (OR: 2.148, 95% CI: 0.439, 3.857) were independently associated with acceptability. Child’s age (OR: -0.108, 95% CI:0.818, 0.985), anemia knowledge (OR:0.016, 95% CI: 1.003, 1.031) and acceptability (OR:0.236, 95% CI:1.140, 1.406) were significant determinants of adherence. Anemia knowledge was significantly associated with the mother’s education and household income when adjusted. In conclusion, unpleasant smell/taste and daily schedule were reported as barriers to MMN use. Yet, perception and trust regarding health benefits were encouraging. Reported adherence was somewhat high. Improving acceptability and anemia knowledge could enhance adherence further in this population.

## Introduction

Micronutrient deficiencies are mostly hidden, and they are clinically less visible [1]. However, vitamin A, iron, and zinc contribute significantly to morbidity and mortality in children under five years of age [2]. In low-income countries, the prevalence of iron deficiency in infants and toddlers ranges from 25–90% [3]. In comparison, in Sri Lanka, it is about 26.5% [4]. In addition, vitamin A deficiency is not uncommon, seen in 30% of children in Sri Lanka [4]. In response to high deficiency rates, the MMN supplementation program for 6–23-month-old children was first launched in Sri Lanka in 2007. The preparation contains 15 micronutrients, including iron (10mg), retinol, zinc, iodine, and vitamins (A, B1, B2, B3, B6, B12, C, D, E, and folic acid), copper, and selenium.

The safety and efficacy of MMN powder are well established [2]. A systematic review of 17 randomized trials had shown that fortification reduces anemia, iron deficiency anemia, and vitamin A deficiency by 34%, 57%, and 21% [5]. The effectiveness of such nutritional interventions is also dependent on the acceptability of the recipients [6]. Previous studies have reported mixed results on acceptability. Many studies have shown reasonably good caregiver satisfaction, attributed to ease of preparation and use and perceived health benefits [7]. In contrast, some studies reported caregiver concerns regarding color change as well as unpleasant taste when nixed with food, in addition to the bitterness of MMN itself [7–9]. The adherence rate is variable, ranged from 31% to 96% [7]. Some authors have reported higher adherence rates with flexible schedules (e.g., non-daily) than with inflexible, daily schedules [10]. Also, regular visits to households by the community health care workers had shown a positive effect on adherence [9].

In Sri Lanka, the current fortification program recommends the daily consumption of MMN. MMN is distributed at the community-based child welfare clinics. In 2012, a post-implementation survey of the previous alternate day schedule reported high adherence and reduced anemia [4]. However, acceptability was not explored in that survey conducted in Sri Lanka [4]. Further, anecdotal reports of unpleasant taste and smell and refusal to eat when MMN is mixed are not uncommon. Thus, the need for more understanding of the adherence and acceptability of the current MMN program was apparent. Therefore, we aimed to study the acceptance and adherence of the current schedule of MMN powder among children aged 7–23 months in an urban community setting in Sri Lanka.

## Materials and methods

### Study design, population, and study setting

It was a community-based, cross-sectional study in primary health units in an urban setting in the Colombo district from May to November 2019. This is an independent study and not part of the nutritional programme monitoring. The community health units coincide with local government administrative units and provide free health services, focusing mainly on maternal and child health. In Sri Lanka, MMN powder is distributed only through community clinics, free of charge. Each child is given 30 sachets per month for two months (60 sachets) at 6, 12, and 18-months. However, the timing of distribution depends on the supplies and clinic schedules. Primary healthcare workers with a background in nursing and/or midwifery are responsible for distributing the MMN sachets and giving health education. The study population included parents of children 7 to 23 months who had received MMN sachets during the previous month. The equation for calculating sample size is as follows:

$$n=Z^{2}\frac{P\;(1-P)}{e^{2}}$$

[Z = level of confidence (1.96), e = margin of error (0.05)]. P was taken as 89% for adherence to MMN, based on a previous study from Sri Lanka in 2012 [4]. The purpose of that study was to assess the need, effectiveness, efficiency, and sustainability of the previous MMN schedule in the 12 intervention districts involving both recipients and non-recipients. The non-response rate was considered as 10%. Thus, the minimum sample size required was 150. Recruitment was done using cluster sampling. Initially, four MOH areas were selected on a convenient basis. Each MOH area has a varying number of multiple satellite clinics. Four to six such clinics were randomly selected from each MOH area. The sample size allocated to each MOH area was proportionate to the population density of that area. Then, the allocated sample size for each MOH area was divided equally among the selected satellite clinics. The purpose of allocating an equal number of participants from randomly selected clinics in each MOH was to increase the validity of the sample by reflecting the characteristics of the population examined. In each clinic, the potential participants were recruited through convenient sampling. The parents/guardians of children 7 to 23 months were screened for inclusion. Initial screening excluded children with chronic diseases and children who had not received MMN for various reasons (unavailability or high-risk/nutrient-deficient children who received supplements from hospital outpatient clinics). All children who fulfilled the inclusion criteria were invited to participate. Incomplete questionnaires were removed from the final analysis. Ethics approval was obtained from the Ethics Review Committee of the Faculty of Medical Sciences, University of Sri Jayewardenepura (B.Ph/03/19).

### Data collection procedure

The self-administered questionnaire was developed based on previous experience [9, 11]. It captured information on socio-demography, acceptability, adherence, and knowledge of anemia and MMN sachets. Content and item construction were checked by two pediatricians who are experts in the field. The questionnaire was pretested on eight respondents for clarity and easiness to answer. A team performed forward and backward translations of the English version and assessed for appropriateness and discrepancies. It was available in three languages (English, Sinhalese, and Tamil). Education was categorized as primary (Grade 1–5), junior secondary (Grade 6–9), senior secondary (Grade 10–11), collegiate (Grade 12–13), and tertiary education [12]. Monthly average household income was categorized as poor/poorest (<36,445 Sri Lankan Rupees-LKR), middle (36,445–81,372 LKR), and richer/richest (>81,372 LKR) [13] and mother’s employment as homemakers or income-generating. The questions assessed anemia knowledge (symptoms and food items etc.) and MMN sachets (contents and administration). The assessment of acceptance was based on four themes; smell and taste, ease of administration, health gains, ease of use, and needs perception. Those questions were answered on a 5-point Likert scale (strongly agree = 5, agree = 4, neither agree nor disagree = 3, disagree = 2, strongly disagree = 1). Additionally, if they disagreed or were neutral about the smell and taste, an open-ended question was given to indicate suggestions/actions for improvement. Adherence was assessed by recalling the number of sachets remaining, followed by a telephone call to confirm if the respondents indicated the need for verification. An open-ended question allowed the respondents to write up reasons for missing sachets if they had. Data collection is done after obtaining written informed consent.

### Data analysis

Data were analyzed by using Statistical Package for Social Science (SPSS) version 22. Data were assessed for skewness and kurtosis. The constructed Cronbach’s reliability score for all ten questions on acceptability was 0.801. The final score for acceptance was calculated and converted to a percentage-a higher score indicated higher acceptability. Adherence to ≥80% sachets of the prescribed number for one month was considered as “adequate adherence” [7, 14]. The adherence rate of the sample population was calculated as the number of children who adhered adequately out of the total number of children who were given MMN during the past month. If sachets were missed due to illness or removed due to spoilage, the calculations were adjusted accordingly.

The section on knowledge on anemia consisted of eight questions. Each answer was given an equal weightage in calculating the score (the total maximum score was 4). To improve the initial Cronbach alpha reliability, we did a factor analysis to decide which component to be removed. Subsequently, Cronbach’s alpha reliability improved to 0.642 with the removal of four questions. This score was subsequently converted to a percentage. A higher percentage indicated a higher knowledge. A score less than 50% was considered as ’poor.’ Knowledge of the contents and method of administration of MMN sachets were tested with two questions, and answers were analyzed separately. The results were expressed as percentages.

Correlations between adherence vs. acceptability score were tested with Spearman correlation analysis as the number of sachets consumed (adherence) did not have a normal distribution. Regression analysis was performed to examine associations. In univariate regression analysis (linear/logistic), adherence (adequate vs. inadequate), acceptability, and anemia knowledge (continuous variables) were included as dependent variables. Independent variables were either continuous (e.g., child’s age, monthly average household income, anemia knowledge) or categorical (e.g., gender, mother’s education, father’s education, and extended family). In multivariate regression analysis, the variables with a p-value of ≤0.25 were included. Odds ratios (OR) were calculated with a 95% confidence interval (CI). Independent variables were checked for multicollinearity, excluded if highly correlated (correlation co-efficient >0.7).

## Results

### Demographic characteristics

A total of 206 children were screened. Twenty-five were not eligible as they had not received MMN sachets due to non-availability. Another sixteen were not included since they were not given MMN and were prescribed vitamin supplements by hospital outpatient clinics/independent practitioners (viz., premature, nutrient-deficient). Thus, a total of 165 eligible caregivers were invited. Three did not consent to participate. Subsequently, nine were excluded due to incomplete questionnaires. A total of 153 records were analyzed. The response rate was (153/165 = 92.7%).

The median age (range) of the children was 12 (7–23) months. Most were ≤12 months (57.5%). The index child was the first child in 46.4%. Most mothers (54.9%) were homemakers. All respondents and their spouses had at least primary education. In 21%, the household income was either in the "poorest" or "poorer" category. The median household income per month was 65,000 LKR (range 18,000–400,000 LKR). The sample characteristics are given in Table 1.

**Table 1:** Distribution of socio-demographic characteristics.

Characteristic	number (%)
Age of the child, Median (range)	12 (7, 23)
Gender of the child (males)	78 (50.3)
Birth order (first child)	71 (46.4)
Number of siblings, median (range)	01 (0, 3)
Extended family (yes)	73 (47.7)
Household income/month (LKR)	
Poor & Poorest (<36,445)	32 (21)
Middle (36,445–81,372)	70 (45.7)
Richer & Richest (>81,372)	51 (33.3)
Mother’s education	
Senior secondary/less	38 (24.8)
Collegiate	45 (29.4)
Tertiary	70 (45.8)
Father’s education	
Senior secondary/less	38 (24.8)
Collegiate	47 (30.7)
Tertiary	68 (44.5)
Mother’s employment	
Homemakers	84 (54.9)
Income-generating	69 (45.1)

### Acceptability of MMN

The mean (SD) acceptability score of the study sample was 67.81% (9.37%). The acceptability score was below the mean in 40.7%. Scores are summarized in Table 2. The highest mean scores were observed for need perception and health benefits. The majority (51%, n = 78) disagreed with the taste or smell, 30% (n = 46) were neutral (“neither agreed nor disagreed”). Also, 47.7% agreed that they had experienced difficult mealtime. Two-thirds expressed their suggestions/actions to overcome the unpleasant taste and smell; changing the dosage form to syrup/liquid (n = 65), tablet/pills to crush and hide in food (n = 4), mix with yogurt or fruit juice or milk or cereal (n = 10), make the product sweeter (n = 9), dividing one sachet between meals/offer at the end of the meal (n = 4) and cannot think of a method (n = 8). One-quarter (25.9%) disagreed with the existing daily schedule, while 29% "neither agreed nor disagreed."

**Table 2:** Mean acceptability scores and percentage agreed/disagreed with each statement.

	Mean score±SD	Mean % score	% Strongly agreed or agreed	% Strongly disagreed/ disagreed
***Smell and taste***				
Child likes taste/smell	2.65±0.87	53.0	18.9	51
*Difficult mealtime when mixed	2.67±0.80	53.4	47.7	15.7
***Health gains***				
Weight improvement	3.65±0.75	73.0	62.8	5.9
Increase in energy level	3.63±0.69	72.6	56.9	2.6
Getting less infections	3.50±0.74	70.0	50.9	6.6
***Ease of use***				
*Prefer flexible schedules	3.20±0.91	64.0	25.5	45.1
Amount in one packet was just fine	3.80±0.62	76.0	79.1	4.0
Storage is easy	3.40±0.91	68.0	61.4	25,5
***Need perception***				
Supplements are essential for growth	3.75±0.68	75.0	76.5	6.6
Like to continue using this product	3.41±0.95	68.2	62.8	25.5

*These items were reverse scored. Likert scale: Strongly agree = 5, agree = 4, neither agree nor disagree = 3, disagree = 2, strongly disagree = 1.

### Adherence to MMN sachets

The adherence rate was 72.5% (percentage of children who consumed ≥80% of prescribed per month). The caregivers reported reasons for missing sachets. Rejected by the child (n = 36), forgot/busy (n = 7) perceived adverse effects (vomiting = 6, constipation = 3, loose stools = 2 and rash = 1), child’s illness (n = 13), spoilage (n = 3), and no perceived requirement for vitamins (n = 3) were cited. The Spearman correlation between adherence and acceptance was significant (r = 0.494, p<0.001).

### Knowledge of anemia and MMN sachets

Knowledge of anemia and MMN sachets is summarized in Table 3. The mean (SD) for anemia knowledge was 64.86% (31.73%). There were (21.9%) participants whose knowledge score was less than 50%. Most identified iron deficiency as the main reason for anemia in young children. However, most (56%) were not aware that red meat is an iron-rich food item. The majority knew that the MMN contains vitamins and minerals. Also, most were aware that it should not be mixed the entire meal.

**Table 3:** Knowledge on anemia and MMN sachets.

	Correctly answered, n	Percentage
Iron deficiency is the commonest cause of anemia in children (yes)	126	82.3
*A common symptom of anemia (lack of energy)	110	71.9
*The food item with the highest amount of iron (red meat)	67	43.8
Iron deficiency anemia is preventable (yes)	94	61.4
*The contents in the MMN sachets (multiple vitamins & minerals)	130	84.9
MMN powder mixed with the entire meal (no)	124	81

*multiple-choice questions, Correct answer is given within brackets.

### Factors associated with acceptability

In univariate analysis, child’s age was a significant predictor of acceptability (Table 4). All variables with a p-value of ≤0.25 were considered for inclusion in the multivariate model (child’s age, birth order, number of siblings, father’s education, and household income). However, the number of siblings was not considered as it had significant collinearity with birth order.

**Table 4:** Univariate analysis of factors affecting acceptability.

Variable		Adjusted OR	95% CI		p-value
			Lower	Upper	
*Child’s Age (months)		-0.376	-0.528	0.224	<0.001
Child’s gender	Female	1			
	Male	0.278	-1.264	1.780	0.715
*Number of siblings		-0.702	-1.694	0.291	0.165
Birth order of the child	First	1			
	≥ Second	1.208	-0.286	2.702	0.112
Extended family	No	1			
	Yes	0.159	-1.665	1.347	0.835
Mother’s education	Tertiary	1			
	Collegiate	0.486	-1.294	2.265	0.590
	Senior secondary/less	0.180	-1.696	2.057	0.850
Father’s education	Tertiary	1			
	Collegiate	-0.341	-2.087	1.404	0.700
	Senior secondary/less	1.567	-0.297	3.430	0.099
Mother’s employment	Homemakers	1			
	Income-generating	-0.191	-1.700	1.318	0.803
*Household income (LKR)		0.009	-0.006	0.024	0.250
*Anemia knowledge		0.008	-0.016	0.031	0.524

*Continuous variables

The final model included only the child’s age, father’s education, and household income in the multivariate logistic regression analysis. Another remaining variable (viz. birth order) did not improve the model. The child’s age and father’s education were independently associated with acceptability (Table 5). As the child’s age increased, acceptability became lower. On the other hand, acceptability was higher among fathers with senior secondary or lower educational attainment than fathers with tertiary education.

**Table 5:** Multivariate analysis of factors affecting acceptability.

		Adjusted OR	95% CI		p-value
Variable			Lower	Upper	
Child’s age		-0.360	-0.510	-0.211	<0.001
Father’s education	Tertiary	1			
	Senior secondary/less	2.148	0.439	3.857	0.014
Household income		0.014	-0.001	0.028	0.070

Overall model: F 3,149 = 10.61; p<0.001; R^2^ = 0.18.

### Factors associated with adherence

In univariate analysis, child’s age, acceptability, and anemia knowledge were significant predictors of adherence as shown in S1 Table in S1 File. All variables with a p-value of ≤0.25 were considered for inclusion in the multivariate model (child’s age, acceptability, household income, and anemia knowledge). Household income did not contribute to the model significantly. As child’s age was not highly correlated (r = -0.320) to acceptability, both variables were included in the model. Finally, the child’s age, anemia knowledge, and acceptability were considered in the model (Table 6). As the acceptability score increased, adherence improved. Adherence was also increased when anemia knowledge increased, but the strength of association was weak. When the child’s age increased, adherence declined.

**Table 6:** Multivariate analysis of factors affecting adherence.

	Adjusted OR	95% CI		p-value
		Lower	Upper	
Age of the child	-0.108	0.818	0.985	0.022
Knowledge on anemia	0.016	1.003	1.031	0.017
Acceptability score	0.236	1.140	1.406	<0.001

Overall model χ^2^(3) = 48.30, *p* = <0.001, Nagelkerke *R*^*2*^: *39*.*2%*.

### Factors associated with knowledge

Factors that are associated with anemia knowledge scores were analyzed with univariate and multivariate linear regression. In the univariate analysis, mother’s and father’s education, mother’s employment, and household income were significant predictors as shown in S2 Table in S1 File. All variables with a p-value of ≤0.25 were considered for inclusion in the multivariate model (mother’s and father’s education, household income, employment of the mother, and extended family or not). Father’s education, mother’s employment, and extended family did not significantly contribute to the model. The final model given in S3 Table in S1 File contained household income and mother’s education as significant predictors. Mothers with tertiary education had better anemia knowledge than mothers with lower educational achievements (collegiate and senior secondary or less).

## Discussion

This study gives insights into acceptance and adherence to daily food fortification with multiple micronutrients in an urban community setting in Sri Lanka. We found that acceptability was low in specific dimensions, particularly smell/taste. Yet, most respondents reported positive health benefits (weight gain, improved energy, and fewer infections), which could have contributed to the relatively higher adherence rate despite the perceived drawbacks.

Mixed reports on the acceptance of sensory qualities (taste/smell) have been published. Inayati et al. reported that the main reasons for irregular consumption of MMN were the perceived bitter taste and the monotonous taste when consumed daily [15]. A study in Bangladesh among 78 children aged 6–59 months reported lower mean acceptability scores for smell and taste properties compared to other dimensions such as ease of use (75% vs. 92%) [9]. In our cohort, the scores were 53% and 69% for the respective categories (taste/smell and ease of use). In contrast, there were fewer concerns on acceptance of MMN in Niger, and the caregivers perceived that MMN was easy to use, had no unpleasant taste or smell, and did not change the taste of the food. Further, in that study, respondents felt that MMN increased the child’s appetite and weight gain [16].

Despite the lower acceptance of taste and smell properties in this study, the majority expressed the desire for continuing MMN powder. The plausible explanation for willingness to continue is the influence of perceived health benefits. Some participants conveyed their ideas to overcome the unpleasant taste/smell. A few hinted that MMN might be mixed with sweet beverage/fluids to avoid the unpleasant taste, probably not realizing that lipid encapsulated powder tends to float on liquids. It also gives a bitter taste when mixed with liquids. Others suggested making the product sweet-flavored or changing the physical form of the supplement (e.g., iron syrup). Yet, previous reports have emphasized the disadvantages of other iron preparations, such as iron syrup and tablets. For example, they give a metallic taste in addition to reported gastrointestinal side effects and teeth staining [17].

The older children rejected the MMN fortification than the younger children. In a growing child, the perception of taste has different stages. When infants grow into early childhood, the preference for sweet taste and the aversion to bitter taste increases [18]. That could be one explanation why toddlers being picky eaters compared to infants [19]. Therefore, taste is an important consideration of a nutritional intervention targeted for different age groups.

In the present study, the schedule was reported as a drawback. The flexibility of the schedule has been demonstrated to increase acceptability and adherence [20]. A cluster-randomized control trial among children aged 6–23 months showed better adherence, acceptability, and hematological response to MMN sprinkles with a flexible schedule over four months than a daily schedule [20]. Higher adherence to flexible schedules than daily administration can be attributed to difficulties remembering or feeding when given daily.

We found that the acceptance was higher among fathers with lower educational levels (≤ grade eleven) than with tertiary education. It might be that individuals with higher qualifications are too selective and fastidious and do not just passively accept interventions as they are. Such issues could probably be resolved if the primary healthcare workers are ready to discuss the importance of the intervention and clear the doubts of the caregivers [21].

In this study, the majority of the children consumed MMN adequately (72.5%). However, variable adherence rates have been documented in the literature (31–96%) [7]. One reason for such a wide variability could have been different definitions used to label "adequate adherence." Another reason could be that the reported adherence may not be reflecting the respondents’ actual behavior. Social desirability bias could have affected the actual behavior, for example, under-reporting non-compliance, as participants were unwilling to react negatively to the questions asked [22].

Overall acceptability influenced the adherence among the respondents. Therefore, targeting the acceptability by improving sensory characteristics and schedule could improve the outcome of the nutritional strategy in this population. Also, anemia knowledge influenced adherence to some extent. Knowledge dissemination is a strategy that could be easily conceivable in a country with a strong primary healthcare model, like Sri Lanka. Knowledge dissemination should be tailored to the socio-cultural background of the target population. Sometimes nutritional supplements may not be adequate to combat iron deficiency. A healthy diet rich in iron (viz. flesh food) is also an important component. However, flesh food consumption is influenced by economic, cultural, and religious factors [23]. The use of low-cost, non-flesh, iron-rich food items should be supported in a resource-poor setup like ours with a predominantly starch-based diet with low flesh food consumption [23]. Low flesh-food consumption could have been why most of the respondents were unaware that red meat is an iron-rich food. We also found that household income was linked to anemia knowledge in addition to the mother’s literacy level. Therefore, we should keep in mind that anemia preventive strategies should also involve improving household income and disseminating knowledge [24].

The findings should be interpreted with the following limitations. First, the study was conducted in an urban setting; thus, the findings do not reflect other settings (e.g., rural). Socioeconomic differences, lower levels of employment, lower educational achievements, service, resource, and transportation-related imbalances between urban and rural/remote areas affect the acceptability and adherence findings [25]. Second, our calculated sample size was small and was not corrected for multi-stage cluster sampling. Larger sample size would have given greater statistical power. Further, we used a self-administered questionnaire. We sensed that some caregivers found it difficult to understand some of the questions. Yet, the absence of an interviewer would have provided the respondent with greater anonymity and made them provide truthful information. Finally, our study did not specifically probe into certain important aspects of administration, e.g., whether the MMN had been consumed with solids or liquids. In addition to the quantitative questionnaire, a mixed-method survey with a qualitative component would have perhaps given more understanding of the topic. Another drawback was that we relied on caregivers’ reports on adherence, which tends to recall bias.

## Conclusions

Unpleasant smell/taste and daily schedule were reported as barriers to MMN use. Yet, perception and trust regarding health benefits were encouraging. Reported adherence was somewhat high. Improving acceptability and anemia knowledge could enhance adherence further in this population. Future surveys should explore the socio-cultural diversities of acceptance, palatable preparations, and convenient schedules in similar settings.

## Supporting information

S1 File (PDF)
