# Supplementary material for: Acceptability of home fortification with multiple micro-nutrients among Sri Lankan children
Source: PLoS One. 2021 Dec 22;16(12):e0261516. doi: 10.1371/journal.pone.0261516 (PMC8694412; doi:10.1371/journal.pone.0261516)
Supplement: S1 File — (PDF) [file pone.0261516.s001.pdf]

Table S1: Univariate analysis of factors affecting adherence

Variable		Adjusted OR	95% CI		p-value
			Lower	Upper	
*Child's age (months)		-0.171	0.777	0.915	<0.001
Child's gender	Female	1			
	Male	-0.077	0.455	1.884	0.831
*Number of siblings		-0.082	0.577	1.471	0.730
Birth order of the child	First				
	≥ Second	0.065	0.523	2.176	0.859
Extended family	No	1			
	Yes	0.332	0.678	2.862	0.367
Mother's education	Tertiary	1			
	Collegiate	0.212	0.526	2.907	0.627
	Senior secondary/less	-0.018	0.411	2.348	0.967
Father's education	Tertiary	1			
	Collegiate	0.130	0.456	2.844	0.780
	Senior secondary/less	20.249	-	-	0.999
Mother's employment	Housewife	1			
	Income generating	0.259	0.631	2.663	0.480
*Household income (LKR)		0.006	0.998	1.015	0.158
*Knowledge on anaemia		0.015	1.004	1.027	0.008
Acceptability		0.269	1.184	1.446	<0.001

*Continuous variables

Table S2: Univariate analysis of factors associated with anemia knowledge

Variable		Adjusted OR	95% CI		p-value
			Lower	Upper	
*Child's age (months)		-0.458	-1.562	0.646	0.413
Child's gender	Female	1			
	Male	-0.256	-10.429	9.916	0.960
*Number of siblings		-2.714	-9.461	4.032	0.428
Birth order of the child	First				
	≥ Second	3.345	-13.617	6.747	0.506

Extended family	No	1			
	Yes	7.077	-3.057	17.210	0.170
Mother's education	Tertiary	1			
	Collegiate	-24.37	-34.55	-14.16	<0.001
	Senior secondary/less	-40.69	-51.43	-29.96	<0.001
Father's education	Tertiary	1			
	Collegiate	-30.23	-40.62	-19.83	<0.001
	Senior secondary/less	-33.01	-44.12	22.91	<0.001
Mother's employment	Housewife	1			
	Income generating	21.752	12.149	31.355	<0.001
*Household income (LKR)		0.271	0.180	0.363	<0.001

*Continuous variables

Table S3: Factors affecting anemia knowledge

Variable		Adjusted OR	95% CI		p-value
			Lower	Upper	
Mother's education	Tertiary	1			
	Collegiate	-18.32	-29.65	-6.98	0.002
	Senior secondary/less	-33.04	-45.54	-20.54	<0.001
*Household income (LKR)		0.120	0.016	0.223	0.024

*Continuous variable. Overall model: F 3,150 =22.46, $p<0.001$; R^2= 0.31
